# In vitro comparison of marginal fit between CAD/CAM hybrid ceramic crowns and prefabricated zirconia crowns for primary molars

**DOI:** 10.1007/s10266-025-01089-2

**Published:** 2025-04-12

**Authors:** Nehal Raid Salman, Nagwa Mohammad Ali Khattab, Yasser Gomaa, Ahmad Abdel Hamid Elheeny

**Affiliations:** 1 Pediatric Dentistry, Faculty of Dentistry, Pharos University, Alexandria, Egypt; 2 Pediatric and Community Dentistry, Faculty of Dentistry, Ain Shams University, Cairo, Egypt; 3 Dental Biomaterials, Faculty of Dentistry, Minia University, Minya, Egypt; 4 Pediatric and Community Dentistry, Faculty of Dentistry, Minia University, Minya, Egypt

**Keywords:** Aesthetic, Child, Vita Enamic crown

## Abstract

This study aimed to compare the marginal fit of the hybrid ceramic Vita Enamic (VE) crown fabricated with CAD/CAM versus prefabricated zirconia crown (PZC). An in vitro study was conducted on fifty epoxy resin replicas of 25 standardly prepared mandibular second primary molars (2 replicas for each tooth) were obtained. One tooth was restored with CAD/CAM VE (Zahnfabrik, H. Rauter GmbH & Co. KG) after an optical impression acquired using CEREC Omnicam and the other tooth restored with PZC (NuSmile, Houston, TX, USA). Specimens were sectioned buccolingually and mesiodistally. The marginal fit average was measured using a scanning electron microscope (SEM). The difference was tested for statistical significance using an independent t test at a 5% significance level and 95% CI. CAD/CAM VE crowns showed superior marginal adaptation over the PZCs with a significant difference. The mean difference at the buccolingual section was –10.52 µm with a 95% CI of –20.99; –0.05 (P = 0.049). At the mesiodistal section, the mean difference was –10.56 µm with a 95% CI of –20.82; –0.30 (P = 0.044).VE provides a superior marginal fit over the PZC in the restoration of primary molars.

## Background

Restoration of decayed primary molars with esthetic restorations has become a concern in pediatric dentistry. Several esthetic full-coverage restorations, such as open-faced preformed metal crowns (PMCs), bonded strip crowns, and pre-veneered PMCs, have emerged as a substitute for poor esthetic PMCs. However, these restorations were associated with some flaws, such as inferior gingival status, restoration margin visibility, and restoration total or partial loss and/or chipping, which all of which contribute to increased costs [1, 2].

Using prefabricated zirconia crowns (PZCs) in the restoration of primary dentition is quite new. Besides the superior esthetic qualities of PZCs, their mechanical strength is higher than that of porcelain-fused-to-metal crowns because they were fabricated from a densely sintered monoblock [1]. At room temperature, zirconia is present in a favorable metastable tetragonal phase which is stabilized by additives such as Yttrium Oxide (Y_2_O_3_). High flexural strength, toughness, high hardness, and chemical resistance are attributed to the addition of stabilizers to the zirconia [3]. However, lack of contouring or crimping, inability to control the shape, and shortage in the color shades are some of the PZCs drawbacks in the primary dentition [4–7].

Computer-aided design and computer-aided manufacturing (CAD/CAM) has been available for dental use since its development by Duret in France in the 1970s (System Duret CAD/CAM) [8]. One of the approaches uses CAD/CAM technology, such as Ceramic CEREC (Sirona Dental Systems). This digital technology provides an excellent tool allowing single visit fabrication of restoration with precise standards [9].

One of the clinical uses of custom-made CAD/CAM primary molar crowns is that the milled pediatric crown could be one of the alternatives for the restoration of deciduous teeth. A CAD/CAM fabricated crown, milled directly in the pediatric clinic, reduces chairside work time and may offer better adaptation to the prepared tooth. Moreover, its size and morphology can be easily adjusted particularly in areas of arch crowding or space deficiency [10].

Vita Enamic (VE) is an innovative polymer-infiltrated ceramic network material (PICN). The prevailing content of PICN is the porous feldspathic ceramic network (86% by weight) which is infiltrated with copolymer (14% by weight) [11]. PICN incorporates the properties of ceramic and composite resin with a modulus of elasticity and flexural strength comparable to the tooth structure. Compared to ceramics, the modulus of elasticity and the flexural strength of PICN are higher, while possessing similar fracture toughness [12, 13]. The hybrid structure of VE is responsible for its unique properties. For instance, (i) it has similar abrasion characteristics to those of tooth dentin; (ii) it has comparable hardness and flexural strength to the tooth dentin, which reduces the risk of wear of an antagonistic tooth, and (iii) having low flexural strength (150–160 MPa) and fracture toughness (1.5 MPa) [14]. These properties make VE a promising material that can be used in the restoration of primary teeth. For the preservation of tooth structure, the material can be milled to a very thin thickness (0.2–0.5 mm) [15].

Marginal fit is one of the critical determinants of the restoration’s clinical success in terms of durability and helping prevent hypersensitivity, recurrent caries, plaque accumulation, and subsequent gingival and periodontal diseases [16]. Some literatures suggest an average marginal range of 50–150 μm [17], while others recommend a range of 200–30 μm as more convenient [18, 19].

The goal of the innovation behind custom-made CAD/CAM pediatric crowns is to conquer the obstacles that face pediatric dentists during clinical usage. It reduces the patient dental chair time [10]. Easy adaptation and design of the crown to fit the prepared tooth, putting into consideration the margins of the tooth and miscellaneous conditions as well as factors like arch length deficiency, teeth misalignment, or malocclusions [10, 20].

The data regarding marginal adaptation and internal fit of PZC in primary molars are scarce [21, 22]. Additionally, no previous literature has focused on using custom-made CAD/CAM hybrid ceramics to restore primary molars. Therefore, the current in vitro trial was conducted to compare the marginal fit of VE versus PZC. The tested null hypothesis assumed that there was no difference between the marginal adaptations of the two crowns.

## Materials and methods

### Specimen selection and disinfection

This study was approved by the Ethical committee of Faculty of Dentistry, Minia University (Reference no. 318/2019). Forty-three human extracted mandibular second primary molars were selected, with twenty-five molars chosen for the laboratory work. Using the t test in G*Power version 3.1.9.4, with an alpha level of 0.05, a power of 80%, and an effect size of 0.89 derived from previous literature comparing microleakage between PZCs and PMCs, the calculated total sample size was 42 [23]. The sample was rounded to 50 to allow equal distribution into two groups (*n *= 25 per group). The selected teeth were sound or with carious occlusal surfaces solely without undermined cusps (i.e., classified as 1.1 or 1.2 according to Mount and Hume classification). Teeth were extracted due to periodontal problems or for orthodontic purposes. A hand scaler was used to eliminate attached soft tissue deposits from the freshly extracted molars. The teeth were disinfected by immersing in a 0.5% chloramine T solution for 24 hours and then stored in distilled water at 4°C until preparation [24]. Carious teeth were restored with composite resin. Teeth specimens were embedded perpendicularly in self-cured acrylic resin (Takilon, WP-Dental, Barmstedt, Germany) up to 2 mm below the cemento-enamel junction (CEJ), in a standard plastic dental study model for primary dentition (Banna Cast Plastic model).

### Crown reduction standards

To standardize the crown preparation of all specimens, a single pediatric dentist with 10 years of experience was responsible for performing the task. Teeth were prepared according to the technical guide instructions for NuSmile, PZCs preparation was as follows: (*i*) Occlusal reduction of 1.5 to 2 mm was performed using a coarse football diamond bur (379-023C-FG, NTI-Kahla GmbH, Germany). The preparation followed the anatomical contour of the occlusal surface; (*ii*) the circumferential axial walls reduction of approximately 0.5 to 1.25mm to ensure a passive fit of the restorations. To implement axial preparation, a coarse tapered diamond bur (858-012C-FG, NTI-Kahla GmbH, Germany) was used; (*iii*) the preparation margin was carefully extended to a smooth feather edge, ending 1 mm beyond the CEJ, using a fine-tapered diamond bur (858-014F-FG, NTI-Kahla GmbH, Germany). (*iv*) Finally, all lines and point angles were smoothed and finished, and the entire preparation was rechecked to ensure the absence of any undercuts or ledges [23].

### Impression and working dies of the prepared crowns

**Fig. 1:**
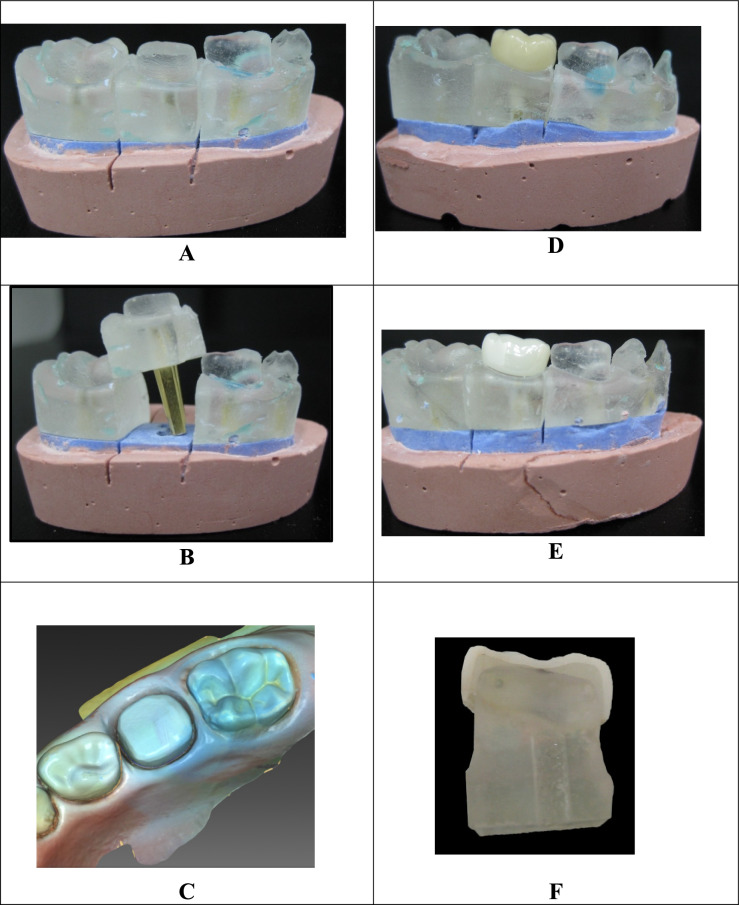
Procedures for groups 1 and 2 **A**: Epoxy resin die of the prepared tooth were fabricated. **B**: A dowel pin was placed at the bottom of the targeted tooth to provide an easy path of insertion and removal. **C**: Biogeneric design technique (CEREC SW 4.6). **D**: CAD/CAM VE crown. **E**: Prefabricated zirconia crown (PZC). **F**: Specimen after being cut with a saw microtome

Two impressions were taken for each prepared tooth using polyvinyl siloxane impression material, to create a pair of molds for each prepared tooth (Speedex Putty, Coltene Whaledent, Altstatten). A quadrafunctional hydrophilic addition reaction silicone elastomeric dental impression material was meticulously syringed onto the prepared teeth, while the remaining impression material was loaded in the impression tray, which had been packed with the putty impression material (Aquasil® Ultra Smart Wetting® Impression material, Dentsply). The impressions were poured with an epoxy resin base and activator (Kemapoxy 150, CMB International), then stored at room temperature for 24 hours. Twenty-five paired negative replicas of the prepared teeth were fabricated and allowed to set. A dowel pin was placed at the base of the targeted tooth to provide an easy path for insertion and removal (Fig .1A, B). An independent investigator was responsible for assigning each pair of prepared teeth to one of two groups. Group “1” (experimental group) had epoxy dies restored with custom-made CAD/CAM crowns, while Group 2 (control group) was restored with PZCs. A total of twenty-five epoxy resin dies were used per group.

### Group “1” (experimental group) custom-made CAD/CAM crowns Optical impression and digitization process

Because of the anatomical similarity between mandibular second primary and permanent molars, a biogeneric design technique (CEREC SW 4.6) was used to set the crown design to the mandibular first permanent molar (Fig. 1C).

Optical impressions of the 25 epoxy resin dies were acquired using a CEREC Omnicam (Dentsply Sirona Dental Systems GmbH, Bensheim, Germany). A full inter-digitation between aligned maxillary and mandibular arches was achieved. The material type was selected in the software. The gingival margin was determined both automatically and manually using the “draw margin” tool, followed by the automatic determination of the crown’s path of insertion. Finally, the occlusal and interproximal contacts were set.

#### Milling process

After creating the digital impression, it was sent via a wireless connection to initiate the milling process of the PICN CAD/CAM PICN block (Vita Enamic Enamic, Zahnfabrik, H. Rauter GmbH & Co. KG, Germany) (shade 2M2-T). The milling was then performed using the inLab milling machine MC XL (Dentsply Sirona Dental systems GmbH, Bensheim, Germany). To simulate the clinical situation of restorations fabricated from VE, the following minimum layer thicknesses must have adhered to: at the bottom of the fissure, at least 1.0 mm; at the cusps, at least 1.5 mm; and circumferentially, between 0.8 and 1.5 mm. The duration of crown designing was two minutes and milling took nine minutes.

#### Specimens polishing

After retrieving the crown from its block, the restoration was hand-polished with Vita Enamic polishing set following the specifications from the manufacturer. The restoration was cleaned with alcohol and dried with oil- and water-free air. The inner surface of the crown was then sandblasted with Al_2_O_3_. Afterward, the roughened surface was salinized with a Vita Enamic. VE Glaze was applied to seal the stains and it was polymerized with all standard bredent's bre.Lux Power unit 2 dental light-curing devices 130W, 100–240 VAC, 50/60Hz with a spectral range of 350– 500 nm. Each crown was then tested for passive seating (Fig. 1D).

#### Cementation and thermocycling process

Each restoration was cemented using G-CEM self-adhesive resin cement (GC Corporation, Tokyo, Japan) according to the manufacturer’s instructions. While the luting cement was setting, an axial load of 2 kilograms was applied using a loading apparatus for 10 minutes to hold the crowns steady in a standardized manner until the cement had fully set. The excess cement was removed with a sharp curette. The specimens were stored in distilled water at room temperature for 4 weeks. Thermocycling was performed on the specimens at a temperatures ranging between 5 ° and 55 °C for 5000 cycles, with a 20-second dwell time [25].

### Group “2” (control group) PZCs

Try-in crowns (pink crowns) were used to accurately determine the appropriate crown size^1^ which was E2. Finally, the PZC was cemented using the same luting cement, technique, and duration that had been adopted in the experimental group 1. For polishing the PZC, a blue zirconia polisher was used at 6000 rpm, followed by a white zirconia polisher (6000 rpm; 94018F 104 055; Komet Dental, Lemgo, Germany) (Fig.1E).

#### Measurement of the marginal gap

**Fig. 2:**
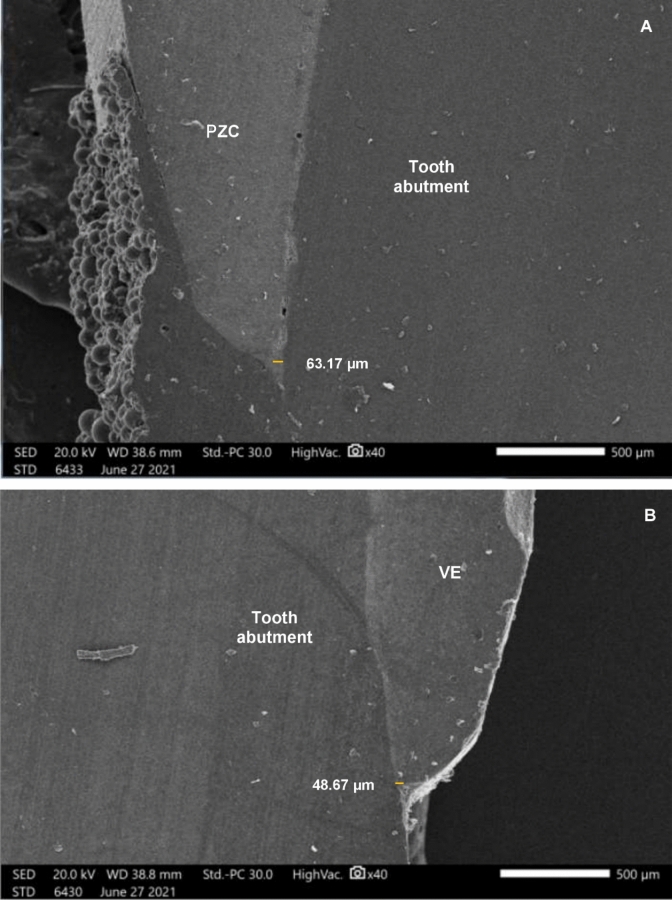
Photomicrographs of marginal gap using SEM (×40) of A=PZC crown, B=VE. Yellow line showing the distance measured from the inner surface of the crown and the prepared tooth at the cervical portion

All specimens were cut axially in two directions (mesiodistally and buccolingually) with a saw microtome (Micracut 150, Metkon Metallography Bursa, Turkey) (Fig.1F). A yellow line was identified at each section to measure the distance from the inner surface of the crown to the prepared tooth at the cervical portion, Fig. 2. The specimens were analyzed with a Scanning Electron Microscope (SEM JSM 200 IT; JEOL 6400, JEOL, Tokyo, Japan). For the SEM evaluation, the exposed interfaces were sequentially polished using a series of silicon carbide abrasive papers with grit sizes of 600, 800, 1,200, 1,500, and 2,000, under running tap water as a lubricant to smooth the surfaces. After rinsing with distilled water, each sample was mounted on stubs and sputter-coated with gold. The samples were then carefully observed under an SEM at the material–crown interfaces. The marginal fit was examined at 100× magnification and internal fit was checked at 40× magnification [26].

#### Statistical data processing

Data were entered into the computer and analyzed using IBM SPSS software package version 20.0. (Armonk, NY: IBM Corp). The Kolmogorov–Smirnov test was used to verify the normality of data distribution. The average marginal gap measurement scores of the two groups were compared using the Student’s *t* test. The significance level was set to 5% and a 95% confidence interval was used.

## Results

Data in Table 1 and Fig. 2 show superior marginal adaptation of the CAD/CAM VE crowns compared to the PZCs, with a significant difference. The mean difference in the buccolingual section was –10.52µm, with a 95% CI of –20.99; –0.05 (*p* = 0.049). In the mesiodistal section, the mean difference was –10.56 µm, with a 95% CI of –20.82; –0.30 (*p* = 0.044).

**Table 1:** Vita Enamic crowns (VE) versus prefabricated zirconia crowns (PZC) mean marginal gap values in micrometers (µm) at the buccolingual and mesiodistal sections

Marginal gap		Group 1 VE	Group 2 PZC	Mean difference	SE	95% *CI*	p value٭
Buccolingual section	Mean ± SD (µm)	53.56 ± (16.80)	64.08 ± (19.89)	–10.52 µm	5.21	–20.99; –0.05	0.049*
Mesiodistal section	Mean ± SD (µm)	49.60 ± (15.56)	60.16 ± (20.22)	–10.56 µm	5.10	–20.82; –0.30	0.044*

95% *CI*: 95-percent confidence interval

*SD* Standard Deviation, *SE* Standard Error

٭Student *t test*; *p* value set to 5%

^*^=statistical significant values

**Fig.3:**
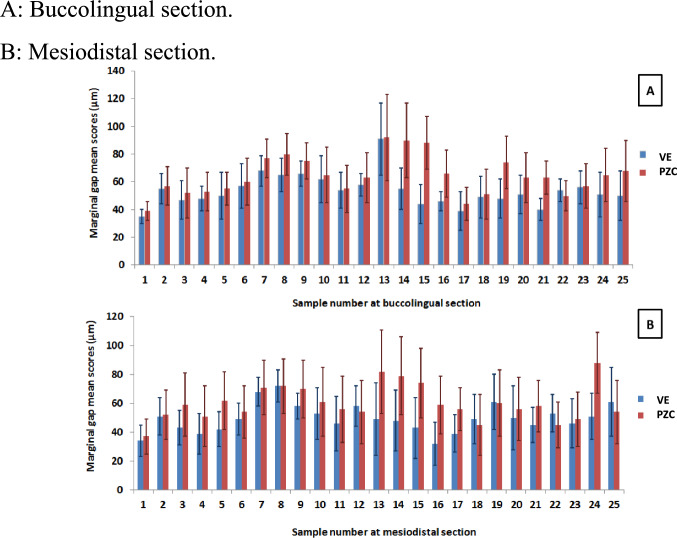
Bar chart showing comparison of marginal mean gap scores between VE and PZC groups. **A**: Buccolingual section. **B**: Mesiodistal section

Fig. 3 shows the average scores of reference points for each specimen of both restorations at the buccolingual and mesiodistal sections. The minimum and the maximum average gap scores for the buccolingual cut (M1) and mesiodistal cut (M2) were as follows: for VE, *M1*=35µm and 9µm, and *M2*=34µm and 72µm. For the PZC, *M1*=39µm and 92µm, and *M2*=37µm and 82µm.

## Discussion

To the best of the author’s knowledge, the current primary laboratory study focused on a newly introduced VE material for primary teeth. This study aimed to compare the marginal fit of a hybrid ceramic material (VE) crown with that of a PZC.

The null hypothesis (*H0*) of the study assumed that there was no difference between the average marginal adaptation values of both restorations. However, the findings of the current study reject this hypothesis. The quantitative assessment of marginal fit for both CAD/CAM crowns was statistically significant.

To mimic the oral environmental status, natural primary molars were used in in vitro studies and embedded 2 mm below the CEJ in epoxy resin to simulate the level of alveolar bone. While natural tooth preparation resembles actual clinical conditions, this study also utilized resin dies to avoid the changeability with extracted teeth. Variance between teeth morphology, time of extraction, thickness, shape, and form complicate the standardization of tooth preparation. Such differences may lead to incompatibility in the cement thickness in the specimens and stress concentration in the crowns, potentially causing fractures [27]. Standardized tooth preparation was performed to accurately control the variables of preparation dimensions. Additionally, thermocycling was used as an aging manipulation to expose specimens to 5,000 thermal cycles, which are equivalent to 6 months of in vivo performance [15, 28–30]. To eliminate the effect of luting cement thickness on the measurement of the internal gap, the same cement was used for both groups under similar occlusal load and thermocycling conditions.

There is no consensus on a standardized method to measure the marginal fit of cemented crowns. In the current study, the cross-sectioning method (CSM) was used to measure the marginal adaptation adopted in several former studies [31–33]. This method permits measuring both the marginal and internal gap using the SEM. The CSM allows accurate calibration of the internal and marginal fit of the restorations [34]. In addition, this approach provides an explicit view of the marginal gap and reduces the possibilities of software or point relocation flaws that may occur with other techniques [33–35]. However, the major drawback is the need for specimen sectioning, making it a destructive method [34].

Other approaches have been accredited to measure the internal fit. For instance, the silicone replica technique (SRT) is a non-destructive, facile, and cost-effective method. However, the elastomeric replica is prone to be destroyed, and faulty sectioning may lead to overestimation of the measurements. Additionally, detecting crown margins and the finishing line can be difficult [36]. Although other 3D digital methods, such as the triple scan method (TSM), micro-computed tomography (MCT), and profile projector, have been increasingly used to assess the fit of prostheses and provide reliable results, a systematic review and meta-analysis conducted by Mai et al, showed no statistically significant difference between digital and conventional methods [37].

Moreover, some shortcomings are encountered in these 3D non-destructive approaches. For example, inaccurate findings can arise from TSM as a result of scanned data overlapping [38, 39]. The use of MCT is less popular than conventional methods, which may be attributed to its unavailability, overprice and technical issues that needs an experienced practitioner, such as extended time of processing and low resolution [40, 41]. Regarding the profile projector method, the main disadvantage is that the luting cement thickness at the margin cannot be directly recorded and must be estimated. Additionally, samples need to be relocated precisely to avert re-profiling inconsistencies [35].

Although the adequate marginal fit is crucial for the long-term performance of restorations, there are no clear, assumed average values for the marginal gap. It is difficult to compare our results with previously published data due to several factor including (i) measurement tools, (ii) preparation design especially primary molars are the spot in this trial, (iii) material type and conditions during the milling process, (iv) size of milling burs, and (v) the luting cement used. However, highlighting some of the prior findings is still helpful.

For instance, CAD/CAM crowns manufactured by CEREC3 or Procera have a marginal gap ranging from 85 to 247 µm [9]. Other studies recommend a mean marginal gap not exceeding 120 µm [42–44]. Another laboratory study showed an average marginal gap of 105 µm for CEREC CAD/CAM crown using the SRT method [38]. Our findings were much less than all of these recorded averages of marginal fit, which indicates an adequate fit of both CAD/CAM restorations. However, the marginal fit of VE was superior to that of PZC.

The mean scores for marginal adaptation of VE in the current laboratory study were less than those of the PZC in both buccolingual and mesiodistal dimensions. Our results were comparable to the findings of Azarbal et al. who reported a mean marginal gap of 47.92±25 µm and 47.92±34.07 µm for 15 specimens restored with VE before and after firing, respectively [45]. Hasanzade et al. compared the marginal fit of three different CAD/CAM materials before and after adjustment with the mean and the SD of adjusted VE margin fit being 50.57±14.75 µm [46].

Regarding the marginal fit of PZCs, Doppalapudi et al showed that PZC had a mean internal gap of 33.25μm ±12.81, which was smaller than that of Figaro crowns when assessed by a stereomicroscope [22]. These results were in conflict with those of the present study due to the different in the test design and the use of extracted human molars. Moreover, a comparable in vitro study by Abo-Elsoud et al, measured the vertical marginal gap of PZC using a digital light microscope, comparing it with Bioflx and PMC groups [47]. Added to that, the results of Abbas et al, showed a significantly larger marginal width measurement for PZCs compared to 3D-printed endocrowns, and they recommended alternatives [48]. A laboratory study compared the marginal discrepancy of 30 CAD/CAM ZC manufactured by two systems; the CEREC inLAB system and the LAVA milling center [49]. They reported an average marginal discrepancy of 53 ± 20μm for the CEREC inLAB system [49]. Another in vitro study showed that the overall mean marginal fit of PZCs for 20 first mandibular molar typodonts with copings fabricated by the CEREC in lab MC XL was 56.9±5.7 μm [50]. The average marginal gap values of these two studies were slightly lower than our findings. These studies reported that zirconia crown is milled directly from solid blocks of zirconia, which allows for better adaptability to the prepared tooth than a PZC [51].

The better results of VE crowns could be attributed to differences in the physical properties of VE and PZC. VE is milled from a softer hybrid ceramic material [45]. Additionally, the milling process of VE crowns involves polishing without exposure to the high temperatures of the sintering cycle. This allows VE crowns to maintain the accuracy of their dimensions to a larger extent [52].

Regarding the clinical relevance of this laboratory study, the availability of CAD/CAM in dental office is becoming increasingly common, offering the advantage of providing precise and esthetic restorations. This enables the delivery of the restorations that meet optimal standards, ensuring maximum benefit for the patients. Despite the high esthetic properties of PZCs, they present some challenges that can be summarized as follows: (*i*) lack of crimping and contouring are the major disadvantages of PZCs in primary dentition [7], (*ii*) aggressive occlusal and circumferential tooth reduction is required to allow passive fit of PZC [53], and (*iii*) the need for passive seating necessitates more aggressive tooth preparation, increasing the risk of pulp exposure in primary dentition [54]. Another important factor for retention of PZCs is crown preparation height. An in vitro study by Jing et al. showed that a minimum of 2 mm of tooth structure is needed for PZC retention [55]. Fourteen studies have evaluated the retention of PZCs; including five RCTs, four observational studies, and five case reports/case series. A total of 427 PZCs were evaluated across all studies, with an 89% retention rate for follow-up periods ranging from 6 to 37 months. Only one study described a significantly low retention rate of 50% [54], (*iv*) there is a lack of long-term clinical research on prefabricated full-coverage zirconia restorations, and (*v*) the use of PZC showed an 11% wear on the opposing dentition [54].

## Study limitations

Although we adopted rigorous measures to achieve a maximum level of standardization, such as identical tooth preparation performed by a single investigator, some concerns need to be considered. For instance, the in vitro trial nature of the trial does not fully mimic the oral environment, and complete control over the parameters cannot be achieved. Thus, in vivo clinical trials are necessary, as they may provide new insights into the desirable marginal gap values for pediatric patients.

Another concern may be related to the use of a 2D measuring instrument, with its previously mentioned weaknesses. Therefore, further in vitro studies are mandatory, using different measuring tools, such as MCT to determine the desired average value for the internal gap in primary teeth.

An advantage of using CAD/CAM block is that they can be used to mill three custom-made crowns, which are financially comparable to PZC without the need for try-ins with pink crowns, thus avoiding additional cost.

## Conclusions

Within the limitations of the study, it can be concluded that:

1. CAD/CAM VE crowns and PZCs have an adequate average marginal gap value.
2. CAD/CAM VE crowns may provide a superior marginal fit over the PZCs in the restoration of primary teeth.

## Data Availability

The datasets generated and/or analyzed during the current study are not publicly available due to privacy concerns but are available from the corresponding author upon reasonable request.

## Abbreviations

VE: Vita Enamic
PZC: Prefabricated zirconia crown
CAD/CAM: Computer-aided design and computer-aided manufacturing
PMCs: Preformed metal crowns
PICN: Polymer-infiltrated ceramic network material (PICN).
SEM: Scanning electron microscope
SRT: Silicone replica technique
TSM: Triple scan method
MCT: Micro-computed tomography

## Footnotes

1 NuSmile, Houston, TX, USA.
